# The American Medical Association

**Published:** 1892-07

**Authors:** 


### *EDITORIAL.*

The office of THE JOURNAL is in rooms 44 and 45 Old Capitol Building.
Address communications relating to the Editorial Department to DR. L. B. GRANDY, Atlanta, Ga., Box 431.
Business communications should be addressed to DR. M. B. HUTCHINS, Atlanta, Ga.
All remittances should be made payable to "ATLANTA MEDICAL AND SURGICAL JOURNAL."
Articles for publication should reach this office not later than the 15th instant.
The editors are not responsible for views expressed by contributors.

### THE AMERICAN MEDICAL ASSOCIATION.

The meeting of this Association held at Detroit, Mich., June 7th, 8th, 9th and 10th, was socially and scientifically one of the most successful in its history. Everything possible was done to make the meeting pleasant and profitable. The profession and laity worked as a unit for the success of the meeting, providing elegant receptions, delightful excursions, etc., but avoiding interference with the work of the Association. Too much cannot be said in praise of Dr. H. O. Walker, of Detroit, Chairman of Committee of Arrangements, for his untiring efforts in behalf of the Association.

The address of President Marcy, of Boston, on "Evolution in Medicine," replete with thought and learning, was received with rapt attention and punctuated with frequent outbursts of applause.

The work of the several sections was, perhaps, better on the whole, than ever before. Especially was this true of the surgical section, under the able chairmanship of our distinguished fellow-townsman, Dr. James McFadden Gaston, to whose untiring energies, faithful devotion and skillful presiding was chiefly due the marked success of this section.

Dr. Gaston's address, before the section, on "Surgery of the

Gall-bladder and Ducts," was a careful, systematic review of the original work of the author, and other surgeons, in this interesting field of surgery.

The unanimity with which the distinguished surgeons who discussed Dr. Gaston's address accorded him the credit of original, pioneer work in this field of surgery must have afforded him great pleasure.

The Association by a unanimous vote urged upon Congress the passage of the bill introduced in the House of Representatives by Mr. Caldwell entitled, "A Bill to Establish a Department of Public Health." Physicians throughout the United States are requested to urge upon their senators and representatives the adoption of the measure.

The Pan American Congress was heartily indorsed by the Association. Dr. Wm. Pepper, President of the Congress, delivered a short address setting forth the scope and expected benefits of the Congress. The Secretary, General Dr. C. A. L. Reed, of Cincinnati, presented his report detailing the progress of the work up to date.

The following preamble and resolutions introduced by Dr. Dudley S. Reynolds, of Kentucky, and which received the unanimous sanction of the Association, without discussion, are of such vital importance to the profession that we print them in full:

"WHEREAS, The Association of American Medical Colleges has adopted the following regulations, viz.: Article III of the Constitution.

Section 1. Members of this Association shall require of all matriculants an English composition in the handwriting of the applicant of not less than two hundred words, an examination by a Committee of the Faculty, or other lawfully constituted Board of Examiners, in higher arithmetic, algebra, elementary physics and Latin prose.

Section 2. Graduates or matriculates of reputable colleges, or high schools of the first grade, or normal schools established by State authority, or those who may have successfully passed the entrance examination provided by the statutes of the State of New York, shall be exempt from the requirements of section 1.

Section 3. Students conditioned in one or more of the branches enumerated as requirements for matriculation shall have time until the beginning of the second year to make up such deficiencies, provided, however, that students who fail in any of the required branches in this second examination shall not be admitted to a record course.

Section 4. Colleges granting final examination on elementary subjects to junior students, shall not issue certificates of such final examination, nor shall any member of this Association confer the degree of Doctor of Medicine upon any person who has not been first examined upon all the branches of the curriculum by the faculty of the College granting the degree.

Section 5. Candidates for the degree of Doctor of Medicine, shall have attended three courses of graded instruction of not less than six months each in three separate years.

Section 6. Students who have matriculated in any regular college prior to July 1, 1892, shall be exempted from these requirements.

*Resolved*, Therefore, that the American Medical Association most heartily endorses the efforts of the Association of American Medical Colleges to advance the cause of medical education, and demands of the medical colleges of the United States the adoption and observance of a standard of requirements which shall in no respect fall below the minimum standard of reqirements adopted by the said college Association."

The following report of the Committee on Railroad Practice was adopted upon motion of Dr. Harvey Reed, of Mansfield, O., Secretary of the National Association of Railway Surgeons:

*To the American Medical Association:*

"The undersigned, members of the committee to which was referred the Memorial of the West Virginia State Medical Society on the Relations of Contract Surgeons of the different Railroad Systems to the General Profession, have given careful attention to the various issues involved in said memorial, and beg leave to report as follows.

The practices specially complained of as reprehensible in said memorial are two.

The first is, that under the rules of the railroad companies, the railroad surgeon is expected to take charge of all employees and passengers injured in railroad accidents, and this without regard to the ethical rights of any outside physician who may have been called in previous to the arrival of the railroad surgeon.

The second is, that the railroad surgeon is in the habit of accepting inadequate compensation for his services, which habit is detrimental to the best interests of the profession by lowering the standard of values for surgical services.

In regard to the first of these complaints the facts are sufficiently simple. The injured passenger or the injured employee may accept the company's physician, in which case the company becomes responsible for the payment of the bill; or he may elect to have some other physician, in which case he must himself undertake to defray the expenses of the treatment. Every injured man has his choice between these two courses and can do just as he pleases about it. The railway company connot force the physician of their choice on the injured man. Why should the injured man have the right to force the physician of his choice on the railway company?

The interests of the railroad company and of the injured man are exactly the same as far as the skillful and successful management of the case is concerned. The man is anxious to recover his wonted health and strength, and the company is equally anxious because of its liability to a suit for damages; and then the greater permanent disability the heavier the damages that will have to be paid. On account of this question of damages the railroads are deeply interested in these cases, and hence their proffer of medical attention to the injured cannot be condemned as an unwarrantable interference. If this proffer may be legitimately made, it may be legitimately accepted, and if the acceptance of it sometimes involves the discharge of the physician first in attendance, that fact furnishes no special ground for complaint. The discharge is the act of the patient, and the patient always has the right to select his medical attendant. The physician first called has charge of the patient just as long as the patient pleases, and no longer. This is all so very plain that further argument does not seem necessary for the settlement of the principle at issue.

In the application of this principle some cases of embarrassment may arise, or even cases of real grievance, and some railroad surgeons may be brusque of manner, and arbitrary and discourteous. Such cases are to be deprecated, but cannot be prevented by any action possible to the American Medical Association.

In regard to the second of these complaints—the complaint that railroad surgeons frequently accept inadequate compensation, it is not needful for us to say much. The question of fees for medical services is a purely local question which every community of doctors must settle for themselves. It is to assert a truism to add, that all underbidding amongst physicians for the sake of getting practice is unethical and unwise, and that any physician who is guilty of it becomes amenable to professional discipline.

In this memorial contract practice is not expressly condemned; but the conclusion is reached that if members of the profession are allowed to make contracts with railroad corporations without detriment to their ethical standing, then all stigma of unprofessional or unethical conduct should be removed from members of the profession who make contracts to do the practice of families.

This question of contract practice is one of great difficulty and importance. We cannot undertake to discuss it with any fullness, but a few words in reference to it may not be amiss.

Before the era of railroads and the rise and multiplication of the great mining and manufacturing industries so characteristic of the present age, contracts to do medical practice were not much known. Indeed, there was little temptation or opportunity for making such contracts, and hence they were regarded by the profession with special disfavor. But times have changed. The great corporations have needed doctors, and the supply has kept pace with the demand. In a word, medical contracts have become very common; and if they are not regarded with special favor, they have won a large amount of professional tolerance. A very cursory survey of the field will show that medical contracts in this country are very numerous—so numerous as to be numbered by tens of thousands.

A very brief capitulation will include the following classes: Medical officers of the Army and Navy and Marine-Hospital Service; medical officers of health, State, county and municipal; medical officers of public and private hospitals, asylums for the insane, and the deaf, dumb and blind; physicians to penitentiaries and jails and almshouses; physicians to military schools and schools not military; medical directors to life insurance companies and beneficiary associations; physicians to mines and factories, and furnaces and workshops; and last but not least, the surgeons who practice by contract for the railroads of the country.

Take the railroad surgeons alone, as they are specially germane to the subject under consideration. According to the best information we can get, the number of railroad surgeons in this country reaches about six thousand (6,000); and the membership of the Association of Railroad Surgeons numbers over twelve hundred (1,200), and is rapidly increasing. At the same time a very large number of the members of the American Medical Association are railroad surgeons. True, all these railroad surgeons are not practicing by contract; but many of them are.

From this brief glance at the statistics of the subject it is evident that contract practice has made too much progress in this country to be crushed out by the adverse resolutions of medical societies. There is no possibility of suppressing it or of making it disreputable.

But while existing facts oblige us to admit all this, a great many of us believe that contract practice has already gone too far, and that it is neither desirable nor wise to extend it to individual and family practice. Too much of the spirit of trade has already found its way into the profession, and its further encroachment should be resisted—not encouraged. Let us continue to maintain, so far as that is yet possible, the old relations of perfect freedom between physicians and patients, with separate compensation for each separate service."

The officers elected for the ensuing year were: President, Dr. Hunter McGuire, Richmond, Va.; Vice Presidents, Drs. H. O. Walker, Michigan; Hawkins Brown, Kentucky; Henry Janes, Vermont; Jesse Hawes, Colorado; Treasurer, R. J. Dunglison; Sec., W. B. Atkinson; Librarian, George W. Webster. Place of meeting, Milwaukee, Wis. Milwaukee is only two hours ride from Chicago, which will give the delegates to next meeting the benefit of the cheap rates to the world's fair without the disadvantage of meeting in an overcrowded city, containing so many things to claim their attention.

No physician who can do so should fail to attend regularly the meetings of the American Medical Association, for aside from the purely medical and scientific work, there is much to be enjoyed which would abundantly compensate him for the outlay in time and money. "The American Medical Association knows no north, no south, no east, no west." We meet together there a band of earnest workers, throwing aside the cares of daily professional routine, but still toiling for our own good and the good of humanity.

